# Oolong tea made from tea plants from different locations in Yunnan and Fujian, China showed similar aroma but different taste characteristics

**DOI:** 10.1186/s40064-016-2229-y

**Published:** 2016-05-10

**Authors:** Chen Wang, Shidong Lv, Yuanshuang Wu, Xuemei Gao, Jiangbing Li, Wenrui Zhang, Qingxiong Meng

**Affiliations:** Faculty of Life Science and Technology, Kunming University of Science and Technology, Kunming, 650500 Yunnan People’s Republic of China; Kunming Grain & Oil and Feed Product Quality Inspection Center, Kunming, 650118 Yunnan People’s Republic of China

**Keywords:** Oolong tea, Main water-soluble contents, Volatile compounds, Processing technology, Aroma characteristics

## Abstract

Consistent aroma characteristics are important for tea products. However, understanding the formation of tea aroma flavor and correspondingly proposing applicable protocols to control tea quality and consistency remain major challenges. Oolong tea is one of the most popular teas with a distinct flavor. Generally, oolong tea is processed with the leaves of tea trees belonging to different subspecies and grown in significantly different regions. In this study, Yunnan and Fujian oolong teas, green tea, black tea, and Pu-erh tea were collected from major tea estates across China. Their sensory evaluation, main water-soluble and volatile compounds were identified and measured. The sensory evaluation, total polysaccharide, caffeine, and catechin content of Yunnan oolong tea was found to be different from that of Fujian oolong tea, a result suggesting that the kinds of tea leaves used in Yunnan and Fujian oolong teas were naturally different. However, according to their aroma compounds, principal component analysis (PCA) and cluster analysis (CA) of the volatile compounds showed that the two types of oolong teas were similar and cannot be clearly distinguished from each other; they are also different from green, black, and Pu-erh teas, a result indicating that the same oolong tea processing technology applied to different tea leaves results in consistent aroma characteristics. The PCA analysis results also indicated that benzylalcohol, indole, safranal, linalool oxides, *β*-ionone, and hexadecanoic acid methyl ester highly contributed to the distinct aroma of oolong tea compared with the other three types of teas. This study proved that the use of the same processing technology on two kinds of tea leaves resulted in a highly consistent tea aroma.

## Background

Oolong tea is a kind of partially fermented tea. It has become one of the most popular beverages in China because of its sweet grassy taste and unique flower-like aroma. Traditional oolong tea is produced with *Camelliasinensis* var. *sinensis* (China type) from Fujian Province in southeast China. However, because of the limited raw tea produced in Fujian Province, tea buds grown in other locations and from different tea tree subspecies have also been used for oolong tea in recent years; an example is *Camellia sinensis* var. *assamica* (Assam type), which is mainly distributed in Yunnan Province in southwest China, especially the districts around Pu-erh (Lv et al. 2014a). These two tea subspecies grown in different locations show obvious differences, such as the sizes of their leaves and their water-soluble components (Liang et al. 2005). Until now, however, little known about the similarities and dissimilarities of these two oolong teas. To determine their consistency in taste, we analyzed the caffeine, catechin, total polysaccharide, and volatile components of Yunnan and Fujian oolong teas and compared them with those of other common types of teas.

Main water-soluble components, such as caffeine, polysaccharides, and catechins (Zhu et al. 2015; Nie et al. 2011), are generally responsible for the taste of tea fusion, whereas volatile components contribute to tea aroma. In tea, volatile components are only present in about 0.01 % of the total dry weight, but they result in a high odor experience because of their low threshold value (Rawat et al. 2007). Whereas water-soluble content is naturally influenced by geographical characteristics, climate, tea cultivar, and processing technology applied on raw leaves, volatile compound content can be influenced and transformed by the processing technology used on the leaves (Fernández-Cáceres et al. 2001; Narukawa et al. 2011). Volatile compounds are transformed from water-soluble components during processing steps, such as fermentation, post-fermentation, and baking (Hara et al. 1995). For example, Yunnan and Fujian oolong teas are both partially fermented by the same processing technology with a series of steps, and they show sweet, fruity, and flower-like odors. Green tea, which is not fermented, has a fresh, grassy flavor. Black tea, which is fully fermented, has a honey, flower-like flavor. Pu-erh tea, which is post-microbially fermented, has a woody and stale flavor (Lv et al. 2015). Whether teas produced from the same types of tea leaves coming from different tea trees show similar or different aroma characteristics has not been extensively studied. For finding the similarity and differences of volatile and water-soluble compounds between Yunnan and Fujian oolong tea, we compared them with those of other kinds of tea to decrease the noises from the data of oolong teas.

In this study, the sensory evaluation, main water-soluble (i.e., caffeine, catechins, and total polysaccharides) and volatile components of Yunnan oolong, Fujian oolong, green, black, and Pu-erh teas were analyzed, and the aroma consistency of oolong teas made from different tea tree leaves was discussed.

## Methods

### Tea samples

Five samples of Yunnan oolong tea were obtained from five typical production sites in Yunnan Province, China and were numbered from YO1 to YO5. Five samples of Fujian oolong tea were also obtained from five typical production sites in Fujian Province, China and were numbered from FO1 to FO5. Ten samples of green tea were likewise collected from Hunan, Yunnan, Sichuan, and Anhui provinces and were numbered from GT1 to GT10, and ten samples of black tea were collected from Yunnan, Anhui, Fujian, and Hunan provinces and were numbered from BT1 to BT10. Finally, 10 samples of Pu-erh tea were collected from Yunnan Province, China and were numbered from CT1 to CT10. In addition, all the tea samples were harvested in spring, 2015; and the varieties of them identified by National Centre for Pu-erh Tea Production Quality Supervision and Inspection, Pu-erh, Yunnan, China.

### Chemicals

The following chemicals and solvents were used: (+)-Catechin (C, ≥ 99 %), (−)-epicatechin (EC, ≥ 98 %), (−)-epigallocatechin (EGC, ≥ 95 %), (−)-epigallocatechin gallate (EGCG, ≥ 95 %), and (−)-epicatechin gallate (ECG, ≥ 98 %) were obtained from Sigma-Aldrich (St. Louis, MO, USA.). Methanol (HPLC grade, ≥ 99.9 %, Lichrosolv, Germany) and acetic acid (HPLC grade, ≥ 99.7 %) were obtained from Fisher Scientific. All other reagents and solvents were of analytical grade and used without further purification, unless otherwise noted. All aqueous solutions were prepared with the use of newly double-distilled water.

### Sensory evaluation

According to the CNIS GB/T 14487-93, three grams of tea sample was extracted with 300 mL of 85 °C distilled water for 15 min. The extracted tea infusion was filtered and cooled to room temperature and then adjusted to a volume of 500 mL. Then the sensory characteristics of the extracted tea infusions were evaluated by five panelists at Faculty of Life Science and Technology, Kunming University of Science and Technology, based on the color, taste and flavor of tea infusions.

### Catechin and caffeine analysis

Samples weighing 0.2 ± 0.001 g were placed in extraction tubes (10 mL). Five milliliters of preheated 70 % water/methanol extraction mixture was filled into each tube individually, incubated in water bath for 10 min at 70 °C, and vortexed for 5 and 10 min, respectively. The extracts were combined and made up to 10 mL with cold methanol/water extraction mixture.

The content and composition of catechins and caffeine in the extract were determined with an HPLC system (2695; Waters Corp., MA, USA) equipped with a Waters Sunfire C_18_ column (5, 4.6 × 250 mm, 35 °C) at 278 nm. The measurement was adjusted as follows: flow rate: 1.0 mL/min; injection volume: 10 μL; mobile phase: A 98 % methanol and 2 % acetic acid, B 98 % water and 2 % acetic acid; gradient elution: 20–25 % A, 0–1 min; 25–45 % A, 1–12 min; 45–90 % A, 12–14.3 min; 90–20 % A, 14.3–15 min; maintained for 5 min. Concentrations of catechins and caffeine were quantified by their peak areas against those of standards prepared from authentic compounds.

### Determination of total polysaccharides

Total polysaccharides were measured according to the method described by Xi et al. (2010). The dry, ground tea leaves (50 g) were extracted with 400 mL distilled water at 90 °C in a water bath for 2 h. After being filtered, the residue was extracted again with 500 mL distilled water for another 2 h. Then, the extracts were centrifuged to remove contaminants. The supernatant was concentrated via rotary evaporation and precipitated with 95 % ethanol. The tea extracts were measured with this method.

### HS-SPME procedure

The HS-SPME parameters of the tea sample were validated and optimized in a previous study (Lv et al. 2014b). Therefore, the same method and parameters were used in the current study to extract the volatile components of the tea samples. Using the same method is advantageous in tracing the change in aroma compounds during the production of the tea sample and in facilitating a comprehensive comparison of the aroma components among four different tea samples. A detailed explanation of the HS-SPME parameters is as follows.

A total of 2.0 g ground tea sample was placed in a 20 mL sealed headspace vial with 5 mL distilled water, and the temperature of the headspace vial was kept at 80 °C for 60 min with an electric hot plate. Then, a 65 μm polydimethylsiloxane/divinylbenzene coating fiber (Supelco Inc., Bellefonte, PA) was exposed to the sample headspace and retained for 60 min. All volatile compounds absorbed on the SPME fiber were desorbed at the GC–MS injector at 250 °C for 3.5 min and then immediately analyzed by GC–MS. After adsorption, SPME coating fiber was transferred to the GC injection port at 250 °C for 30 min.

### GC–MS analysis

An HP 7890A GC instrument combined with an HP 5975C mass selective detector (MSD) quadrupole MS instrument (Agilent Technologies, Palo Alto, CA, USA) was used for the GC–MS analysis. The capillary column utilized was HP-5MS (30 m × 0.25 mm × 0.25 μm film thickness) from Agilent technologies, and high-purity helium (purity 99.999 %) was used as carrier gas at a flow rate of 1 mL/min. The injector and ion source temperatures were set at 250 and 200 °C, respectively. Samples were injected in splitless mode. The initial GC oven temperature was 50 °C, held for 5 min, and then ramped at 3 °C/min to 210 °C, held for 3 min, and finally programmed to 230 °C at 15 °C/min. The Agilent 5975C MS was operated in the electron impact mode using ionization energy of 70 eV with an ionization source temperature of 230 °C and a quadrupole set of 150 °C. The acquisition mode was full scan (from 30 to 500 m/z), and the solvent delay time was 2.8 min.

### Compound identification

With the use of the MSD G1701EA E.02.00.493 chemical workstation data processing system (Agilent Technologies, Palo Alto, CA, USA), peak identifications were made via a search of the National Institute of Standards and Technology (NIST) 08.L MS data library (Qiao et al. 2008; Schuh and Schieberle 2006). The relative percentage content of the aroma components was determined by peak area normalization.

The relative proportions of the constituents were obtained by peak area normalization. Quantitative results were obtained by using the method as follows:

$${\text{Relative content}} \,\left( \% \right) = {{\text{single constituent area}} \mathord{\left/ {\vphantom {{\text{single constituent area}} {\text{total area}}}} \right. \kern-0pt} {\text{total area}}} \times 100\,\%$$

### Data analysis

Significant differences between four different types of tea samples for each of the aroma compounds were determined by Duncan’s multiple range test analysis using SPSS statistical package (version 17.0 for Windows, SPSS, Inc., Chicago, IL, USA). PCA and CA were performed with SIMCA-P software (version 12.0, Umetrics, Umea, Sweden).

## Results and discussion

### Sensory evaluation

**Fig. 1:**
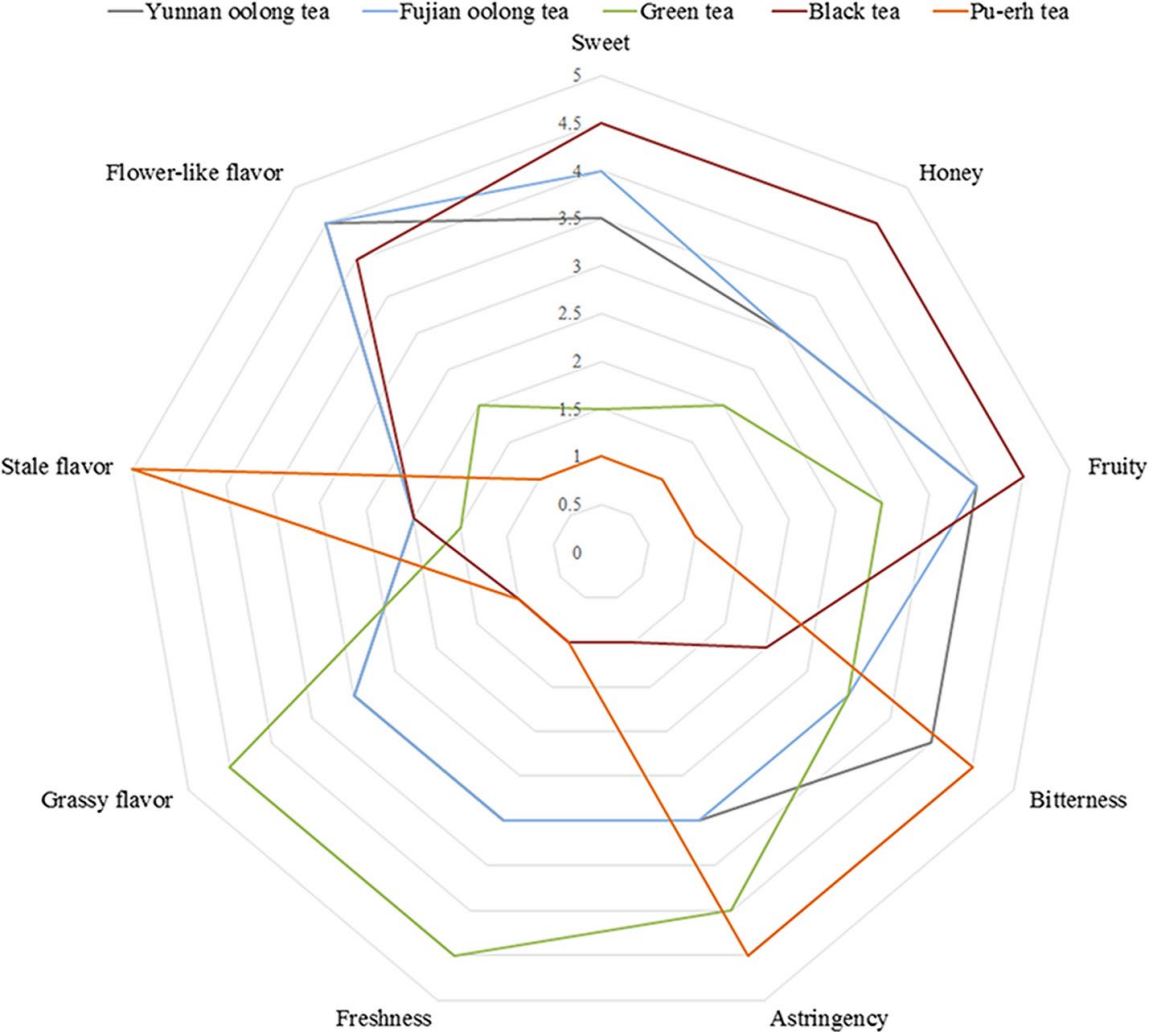
Spider diagram of the sensory evaluation

**Fig. 2:**
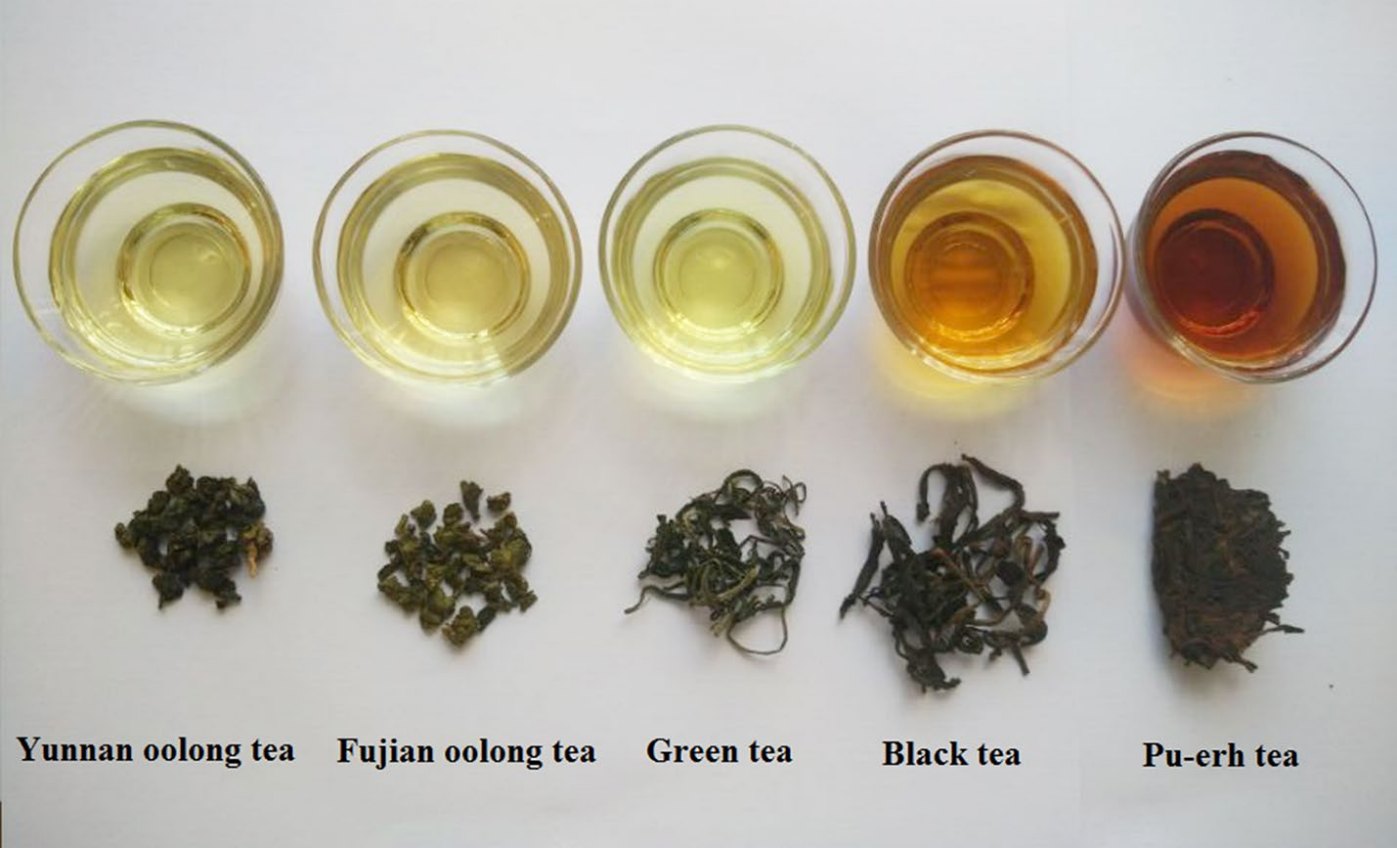
The* shapes* and tea soup color of different types of tea

Sensory evaluation of extracted tea infusions was performed in this work. As shown in Fig. 1, the following scales were used to rank the intensity of these nine attributes: very strong-5.0, strong-4.0, fairly strong-3.0, weak-2.0, very weak-1.0. The results showed that the sensory quality of Yunnan and Fujian oolong teas both were flower-like flavor and sweet, fruity taste; but Yunnan oolong tea infusion showed more bitterness and less sweet than Fujian oolong tea; green tea infusions showed grassy flavor and fresh taste; Black tea has a fruity, flower-like flavor and sweet, honey taste; and Pu-erh tea has a woody, stale flavor and the taste of slight bitterness and astringency. In addition, Fig. 2 showed the differences among the color of Yunnan oolong tea, Fujian oolong tea and other kinds of tea infusions. The color of Pu-erh tea infusion was darkest while that of Yunnan oolong tea infusion was lightest.

### Analysis of the main water-soluble components of Fujian oolong tea, Yunnan oolong tea, green tea, black tea, and Pu-erh tea

Polysaccharides, caffeine, and catechins, which are highly soluble in water, in tea leaf shoots play a significant role in tea quality (Willson and Clifford 1992). Table 1 shows that the caffeine, catechin, and total polysaccharide content of Yunnan and Fujian oolong teas was different (P < 0.05); green, black, and Pu-erh teas had a higher caffeine content than oolong tea (P < 0.05); green tea had the highest catechin content among the five types of teas (P < 0.05), whereas black tea had the highest polysaccharide content. After being semi-fermented, most of the oolong teas, including the Yunnan and Fujian oolong teas, had little catechin content, and their polysaccharide content decreased as well. Yunnan oolong tea had the lowest polysaccharide content among the five types of teas (P < 0.05). Our results were consistent with Wang et al. (2000); Xi et al. (2010); Wang et al. (2011). The findings indicated that *Camelliasinensis* var. *sinensis* and var. *assamica* of the oolong tea samples, i.e., Fujian and Yunnan oolong teas, respectively, were naturally different.

**Table 1:** Total polysaccharides and catechins contents (mg g^−1^) in Yunnan oolong teas, Fujian oolong tea, Green teas, Black teas, and Pu-erh teas

Compound	Yunnan oolong tea (n = 5)	Fujian oolong tea (n = 5)	Green tea (n = 10)	Black tea (n = 10)	Pu-erh tea (n = 10)
EGC	10.31 ± 2.54a^*^	14.15 ± 3.80b	13.90 ± 5.51b	0.37 ± 0.20c	1.19 ± 0.24c
C	2.46 ± 0.94a	4.84 ± 1.12b	6.29 ± 2.36b	1.04 ± 0.69a	1.96 ± 0.41a
EC	2.15 ± 0.85a	4.29 ± 0.65b	5.82 ± 2.22c	1.38 ± 0.73a	1.36 ± 0.48a
EGCG	31.87 ± 8.35a	38.24 ± 8.75b	50.56 ± 8.04c	3.43 ± 1.02d	0.13 ± 0.08d
ECG	4.07 ± 0.69a	8.24 ± 2.68b	17.61 ± 3.39c	3.53 ± 1.40a	0.18 ± 0.16d
Total polysaccharides	14.00 ± 2.41a	18.52 ± 1.53c	10.31 ± 1.50b	18.33 ± 2.47c	17.54 ± 1.73c
Caffeine	14.56 ± 3.27a	16.20 ± 5.48b	26.53 ± 7.65c	21.17 ± 2.72d	22.61 ± 5.8d

*EGC* (−)-epigallocatechin, *C* (+)-catechin, *EC* (−)-epicatechin, *EGCG* (−)-epigallocatechin gallate, *ECG* (−)-epicatechin gallate

^*^For each parameter, different letters within a row indicate difference between different types of tea with Duncan’s multiple range test (P < 0.05)

### Analysis of the volatile compounds of Fujian oolong tea, Yunnan oolong tea, green tea, black tea, and Pu-erh tea

Table 2 shows that a total of 92 aroma compounds were identified in all 40 tea samples. No significant difference between the most volatile compounds of Yunnan oolong tea and Fujian oolong tea was observed (P > 0.05). To differentiate oolong tea from other types of teas, 1-hexanol content served as a valuable index (P < 0.05). Compared with those in other types of teas, benzylalcohol, indole, safranal, linalool oxides, *β*-ionone, and hexadecanoic acid methyl ester were the volatile compounds detected in most of the oolong tea samples (Table 2). These compounds are possibly principal contributors to the fragrant flowery aroma of oolong tea. Their abundant concentrations in oolong tea might be formed during tea manufacture, in which the hydrolysis of their glycosidase and primeverosides by *β*-glucosidase is intensive (Wang et al. 2001). However, some differences were still observed in the volatile compound content of Yunnan and Fujian oolong teas. The 1-Pentanol and 1-octen-3-ol content of Fujian oolong tea was higher than that of Yunnan oolong tea (P < 0.05), whereas the benzaldehyde content of Yunnan oolong tea was higher than that of Fujian oolong tea. These subtle differences should be related to the natural differences of the tea leaves used, as observed in the water-soluble components. Because the most volatile compounds are transformed during fermentation or processing, hypothesizing that these minor differences can mostly be eliminated by the adjustment of processing conditions is reasonable. Generally, the fermentation degree of oolong tea is between that of green and black tea. Therefore, more complicated patterns of aroma flavors can be observed in semi-fermented oolong tea than in unfermented green or fully fermented black tea.

**Table 2:** Volatile components and their relative contents in Yunnan oolong teas, Fujian oolong tea, Green teas, Black teas, and Pu-erh teas

No.	Retention time	Compound	Yunnan oolong tea (n = 5)	Fujian oolong tea (n = 5)	Green tea (n = 10)	Black tea (n = 10)	Pu-erh tea (n = 10)
1	4.140	Hexanal	0.00a^*^	0.00a	0.00a	0.23 ± 0.12b	0.00a
2	5.733	(E)-2-Hexenal	0.00a	0.00a	0.00a	0.1 ± 0.09b	0.00a
3	5.746	cis-3-Hexen-1-ol	0.00a	0.00a	0.00a	0.27 ± 0.23b	0.00a
4	6.260	cis-2-Hexen-1-ol	0.00a	0.00a	0.00a	0.08 ± 0.15a	0.00a
5	6.345	1-Pentanol	0.41 ± 0.67a	1.24 ± 1.01b	0.11 ± 0.17a	0.15 ± 0.17a	0.00a
6	6.741	1-Hexanol	0.18 ± 0.24b	0.33 ± 0.39b	0.00a	0.00a	0.00a
7	7.131	2-Heptanone	0.09 ± 0.13b	0.02 ± 0.05a	0.00a	0.00a	0.00a
8	7.579	2-Heptanol	0.17 ± 0.18b	0.15 ± 0.15ab	0.00a	0.10 ± 0.20ab	0.00a
9	9.989	Benzaldehyde	0.74 ± 0.87b	0.17 ± 0.16a	0.19 ± 0.04a	0.41 ± 0.17ab	0.18 ± 0.08a
10	11.051	1-Octen-3-ol	0.34 ± 0.57ab	0.96 ± 0.48c	0.76 ± 0.78bc	0.11 ± 0.24a	0.03 ± 0.05a
11	11.342	6-Methyl-5-hepten-2-one	0.00a	0.00a	0.25 ± 0.14b	0.00a	0.00a
12	11.599	2-Pentyl-furan	0.00a	0.00a	0.81 ± 0.40bc	1.37 ± 1.12c	0.18 ± 0.10ab
13	13.321	Benzyl alcohol	2.35 ± 1.43c	3.45 ± 0.53d	1.26 ± 0.87b	0.39 ± 0.36a	0.04 ± 0.06a
14	13.590	D-Limonene	1.51 ± 1.60c	1.2 ± 0.10bc	0.32 ± 0.16a	0.59 ± 0.58ab	0.02 ± 0.05a
15	14.049	Phenylacetaldehyde	0.00a	0.00a	0.00a	0.95 ± 0.51b	0.04 ± 0.07a
16	14.123	1H-Pyrrole-2-carboxaldehyde	0.00a	0.00a	0.00a	0.27 ± 0.29b	0.19 ± 0.18ab
17	14.413	Ocimene	0.57 ± 0.52b	0.37 ± 0.25ab	0.43 ± 0.2ab	0.55 ± 0.77b	0.00a
18	15.427	(E)-2-Octen-1-ol	0.00a	0.00a	0.35 ± 0.32b	0.00a	0.00a
19	15.569	Linalool oxide I	3.77 ± 1.44a	4.12 ± 0.83a	0.98 ± 0.57b	1.6 ± 0.85b	1.11 ± 0.64b
20	16.344	Linalool oxide II	4.61 ± 1.92a	4.49 ± 1.15a	2.10 ± 0.77b	3.71 ± 1.77a	2.17 ± 0.85b
21	17.097	Linalool	19.97 ± 2.73a	20.36 ± 1.54a	13.23 ± 4.59a	12.60 ± 14.78a	0.80 ± 0.69b
22	17.23	3,7-Dimethyl-1,5,7-octatriene-3-ol	0.00a	0.00a	0.00a	1.11 ± 1.42b	0.00a
23	17.513	Phenylethyl alcohol	1.86 ± 1.53a	1.15 ± 1.10a	0.41 ± 0.58a	3.85 ± 6.04a	0.36 ± 0.29a
24	19.401	1,2-dimethoxy benzene	0.00a	0.00a	0.00a	0.00a	1.38 ± 0.41b
25	20.266	Linalool oxide III	0.28 ± 0.29ab	0.24 ± 0.11ab	0.00a	0.45 ± 0.45b	0.52 ± 0.29b
26	20.544	Linalool oxide IV	4.64 ± 2.52a	1.35 ± 0.88b	0.70 ± 0.43b	1.88 ± 1.10b	1.71 ± 0.98b
27	20.703	Naphthalene	1.07 ± 1.02a	0.96 ± 0.94a	0.42 ± 0.22ab	0.09 ± 0.13ab	0.51 ± 0.90a
28	21.302	α-Terpineol	2.14 ± 3.39ab	1.65 ± 1.22ab	2.82 ± 1.59b	0.38 ± 0.37a	1.60 ± 0.81ab
29	21.439	Methyl salicylate	2.27 ± 1.84bc	1.89 ± 1.28abc	0.83 ± 1.14ab	3.45 ± 2.00c	0.41 ± 0.31a
30	21.686	Safranal	0.94 ± 0.22a	0.47 ± 0.37b	0.34 ± 0.09b	0.11 ± 0.09c	0.15 ± 0.12c
31	21.85	Dodecane	0.00a	0.00a	2.39 ± 1.46b	0.17 ± 0.32a	0.05 ± 0.08a
32	22.262	Decanal	0.00a	0.00a	0.00a	0.12 ± 0.07a	0.27 ± 0.19b
33	22.672	β-Cyclocitral	0.00a	0.00a	0.63 ± 0.23ab	0.29 ± 0.26b	0.13 ± 0.10c
34	23.135	Nerol	0.91 ± 1.00bc	1.08 ± 1.06c	0.32 ± 0.13a	0.41 ± 0.21ab	0.02 ± 0.06a
35	23.824	3,4-Dimethoxytoluene	0.00a	0.00a	0.00a	0.00a	0.84 ± 0.79b
36	24.467	Geraniol	2.82 ± 2.23a	0.54 ± 0.40a	1.68 ± 0.55a	12.63 ± 7.26b	0.47 ± 0.27a
37	25.293	2-Phenyl-2-butenal	0.00a	0.00a	0.00a	0.38 ± 0.21b	0.00a
38	25.857	2-Methyl-naphthalene	0.00a	0.00a	0.36 ± 0.11c	0.06 ± 0.07a	0.22 ± 0.09b
39	26.004	Indole	0.58 ± 0.47a	0.76 ± 0.29a	0.04 ± 0.09b	0.02 ± 0.07b	0.00b
40	26.475	Tridecane	0.00a	0.00a	6.23 ± 3.66b	0.45 ± 1.07a	0.00a
41	26.578	1-Methylnaphthalene	0.00a	0.00a	0.00a	0.06 ± 0.11a	0.18 ± 0.12b
42	27.027	1,2,3-Trimethoxybenzene	0.00a	0.00a	0.34 ± 0.27a	0.00a	14.41 ± 5.48b
43	27.695	4-Ethyl-1,4-dimethoxybenzene	0.00a	0.00a	0.00a	0.00a	2.3 ± 1.31b
44	28.624	2,6-Dimethoxyphenol	0.47 ± 0.15a	0.79 ± 0.53b	0.35 ± 0.23a	0.24 ± 0.15a	0.31 ± 0.2a
45	29.840	1,2,4-Trimethoxybenzene	0.00a	0.00a	0.00a	0.00a	5.16 ± 2.85b
46	30.093	Damascenone	0.58 ± 0.79a	0.44 ± 0.25a	0.00b	0.21 ± 0.40ab	0.00b
47	30.239	cis-3-Hexen-1-yl Hexanoate	0.00a	0.00a	0.00a	0.46 ± 0.62b	0.00a
48	30.466	Hexyl hexanoate	0.00a	0.00a	0.00a	0.16 ± 0.14b	0.00a
49	30.705	cis-Jasmone	0.81 ± 0.99a	0.57 ± 0.17a	0.48 ± 0.38a	0.63 ± 0.31a	0.39 ± 0.18a
50	30.842	Tetradecane	0.00a	0.00a	1.10 ± 0.26c	0.41 ± 0.10b	0.41 ± 0.12b
51	31.202	1,3,5-Trimethoxybenzene	0.00a	0.00a	0.00a	0.00a	3.40 ± 2.78b
52	31.374	α-Calacorene	0.00a	0.00a	0.05 ± 0.10a	0.20a ± 0.26	0.8 ± 0.4b
53	31.51	β-Caryophyllene	2.81 ± 3.22a	3.08 ± 2.12a	0.54 ± 0.56b	0.06 ± 0.13b	0.00b
54	31.934	α-Ionone	0.81 ± 0.76ab	0.84 ± 0.52ab	1.35 ± 0.48b	0.53 ± 0.41a	0.82 ± 0.31ab
55	32.294	1,2-Benzopyrone	0.00a	0.00a	0.45 ± 0.13c	0.25 ± 0.24b	0.00a
56	32.568	4-(2,6,6-trimethyl-1-cyclohexen-1-yl)butan-2-one	0.00a	0.00a	0.00a	0.03 ± 0.09a	0.14 ± 0.15b
57	32.645	1-Methoxynaphthalene	0.00a	0.00a	0.00a	0.00a	0.58 ± 0.29b
58	32.855	2-Methoxynaphthalene	0.00a	0.00a	0.00a	0.00a	0.71 ± 0.25b
59	32.979	1,2,3,4-Tetramethoxybenzene	0.00a	0.00a	0.00a	0.00a	0.93 ± 0.45b
60	33.039	Geranyl acetone	1.02 ± 1.19a	1.45 ± 0.71ab	2.27 ± 0.82b	1.12 ± 0.53a	1.51 ± 0.68ab
61	33.395	β-Ionone	1.40 ± 1.05b	2.65 ± 1.57c	0.07 ± 0.22a	0.47 ± 0.42a	0.00a
62	34.388	1-(2,6,6-trimethyl-3-cyclohexen-1-yl)-2-buten-1-one	0.00a	0.00a	0.00a	0.15 ± 0.11a	2.91 ± 2.30b
63	34.358	(E)-β-Farnesene	3.93 ± 3.61a	3.49 ± 3.15a	5.34 ± 1.53a	3.35 ± 2.57a	2.67 ± 0.89a
64	34.705	Cocal	0.00a	0.00a	0.00a	0.19 ± 0.19b	0.00a
65	34.97	Pentadecane	1.55 ± 1.78a	1.75 ± 1.11a	0.68 ± 0.21b	0.52 ± 0.12b	0.6 ± 0.16b
66	35.012	Methyl isoeugenol	0.00a	0.00a	0.00a	0.00a	0.41 ± 0.30b
67	35.219	Dibenzofuran	0.00a	0.00a	0.75 ± 0.64b	0.31 ± 0.57ab	0.53 ± 0.35ab
68	35.313	α-Farnesene	0.00a	0.00a	1.13 ± 1.02b	1.11 ± 0.67b	0.32 ± 0.40a
69	35.925	Dihydroactinidiolide	3.81 ± 0.82ab	3.71 ± 0.29ab	6.46 ± 1.12c	2.50 ± 1.29a	4.34 ± 1.48b
70	37.467	Nerolidol	2.86 ± 3.28bc	4.33 ± 1.56c	0.29 ± 0.39a	3.66 ± 1.73c	1.17 ± 0.80ab
71	37.688	cis-3-Hexen-1-yl benzoate	0.00a	0.00a	0.00a	0.66 ± 0.63b	0.00a
72	37.758	Fluorene	0.00a	0.00a	1.07 ± 0.42a	0.14 ± 0.22b	0.70 ± 0.31c
73	38.79	Cedrol	0.00a	0.00a	0.91 ± 0.41b	0.64 ± 1.05ab	1.15 ± 0.87b
74	39.884	Hexadecane	0.53 ± 0.76a	0.83 ± 0.27ab	1.24 ± 0.65bc	0.97 ± 0.23ab	1.64 ± 0.66c
75	40.845	α-Cadinol	0.00a	0.00a	1.17 ± 0.20c	0.51 ± 0.34b	1.07 ± 0.14c
76	40.897	Methyl jasmonate	0.00a	0.00a	0.00a	0.16 ± 0.36a	0.00a
77	41.051	2,2′,5,5′-Tetra methylbiphenyl	0.00a	0.00a	0.00a	0.22 ± 0.21a	0.52 ± 0.39b
78	42.584	Heptadecane	0.91 ± 0.95ab	0.36 ± 0.28a	1.19 ± 0.76ab	0.70 ± 0.46ab	1.44 ± 0.87b
79	42.811	2,6,10,14-Tetramethyl pentadecane	0.00a	0.00a	2.53 ± 1.44c	1.23 ± 0.52b	2.27 ± 1.28bc
80	44.879	Anthracene	0.00a	0.00a	1.17 ± 0.78b	0.88 ± 1.28a	1.39 ± 0.48b
81	46.099	Octadecane	0.84 ± 0.67ab	1.25 ± 0.20b	0.79 ± 0.55ab	0.36 ± 0.26a	1.26 ± 1.01b
82	46.425	2,6,10,14-Tetramethyl hexadecane	0.00a	0.00a	0.61 ± 0.56a	0.49 ± 0.41a	1.44 ± 1.02b
83	47.461	Caffeine	4.27 ± 2.15a	3.59 ± 1.27a	4.44 ± 3.62a	4.50 ± 2.55a	3.88 ± 2.29a
84	47.645	Phytone	2.44 ± 1.51a	2.38 ± 1.58a	3.68 ± 1.47a	3.18 ± 4.64a	3.86 ± 1.83a
85	50.021	Farnesyl acetone	1.49 ± 1.40a	2.15 ± 0.20a	3.04 ± 5.18a	0.18 ± 0.23a	0.49 ± 0.44a
86	50.33	Isophytol	0.45 ± 0.47a	0.31 ± 0.50a	0.16 ± 0.28a	0.17 ± 0.14a	1.25 ± 0.60b
87	51.006	Hexadecanoic acid methyl ester	3.08 ± 1.16a	3.3 ± 0.80a	0.53 ± 0.82c	1.41 ± 0.89b	0.54 ± 0.19c
88	51.657	Hexadecanoic acid	2.25 ± 2.44a	2.44 ± 2.09a	2.36 ± 1.90a	4.7 ± 5.02a	9.05 ± 4.07b
89	52.877	Eicosane	0.00a	0.00a	0.00a	0.00a	0.24 ± 0.23b
90	55.562	Methyl linoleate	0.52 ± 0.49a	0.39 ± 0.25ab	0.19 ± 0.23bc	0.32 ± 0.28abc	0.05 ± 0.11c
91	55.759	Methyl linolenate	0.58 ± 0.55a	0.92 ± 0.61a	0.64 ± 0.63a	0.49 ± 0.38a	0.41 ± 0.31a
92	56.192	Phytol	6.86 ± 2.32a	6.67 ± 2.47a	4.00 ± 3.40ab	4.23 ± 3.53ab	2.10 ± 1.86b

^*^For each parameter, different letters within a row indicate difference between different types of tea with Duncan’s multiple range test (P < 0.05)

**Fig. 3:**
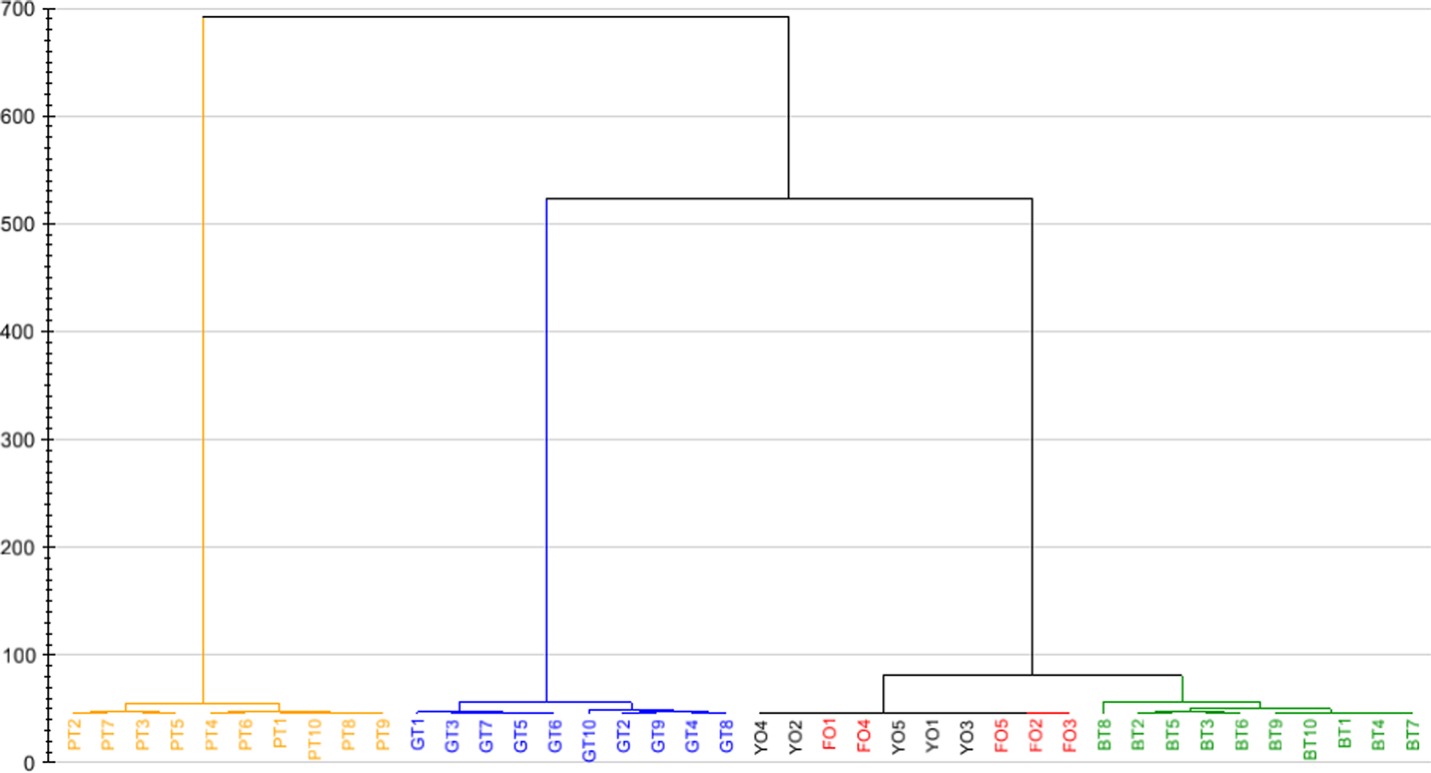
Cluster analysis (CA) dendrogram of 40 tea samples

CA can be used to show the natural groups that exist in a data set on the basis of the information provided by the measured variables (Chen et al. 2008; Wu et al. 2012). All percentage quantitative data of the 92 volatile compounds were used to calculate the CA model. The similarity or diversity between different samples (objects) is usually represented in a dendrogram for ease of explanation. The objects in the same group are similar to one another, and they are different from the objects in other groups. Figure 3 shows that distinguishing Yunnan oolong tea (YO1–YO5) from Fujian oolong tea (FO1–FO5) is difficult; on the other hand, oolong tea (YO1–YO5 and FO1–FO5) and other types of teas (GT1–GT10, BT1–BT10, and PT1–PT10) were clearly different from one another. Oolong tea (YT1–YT5 and FT1–FT5) was clustered more closely with the black tea (BT1–BT10) because they are processed with a fermentation step, although oolong tea was semi-fermented. Finally, the following four main clusters were observed: the first one was composed of ten Pu-erh teas; the second one, ten green teas; the third one, ten oolong teas (five Yunnan oolong teas and five Fujian oolong teas but mixed together); and the fourth one, ten black teas.

**Fig. 4:**
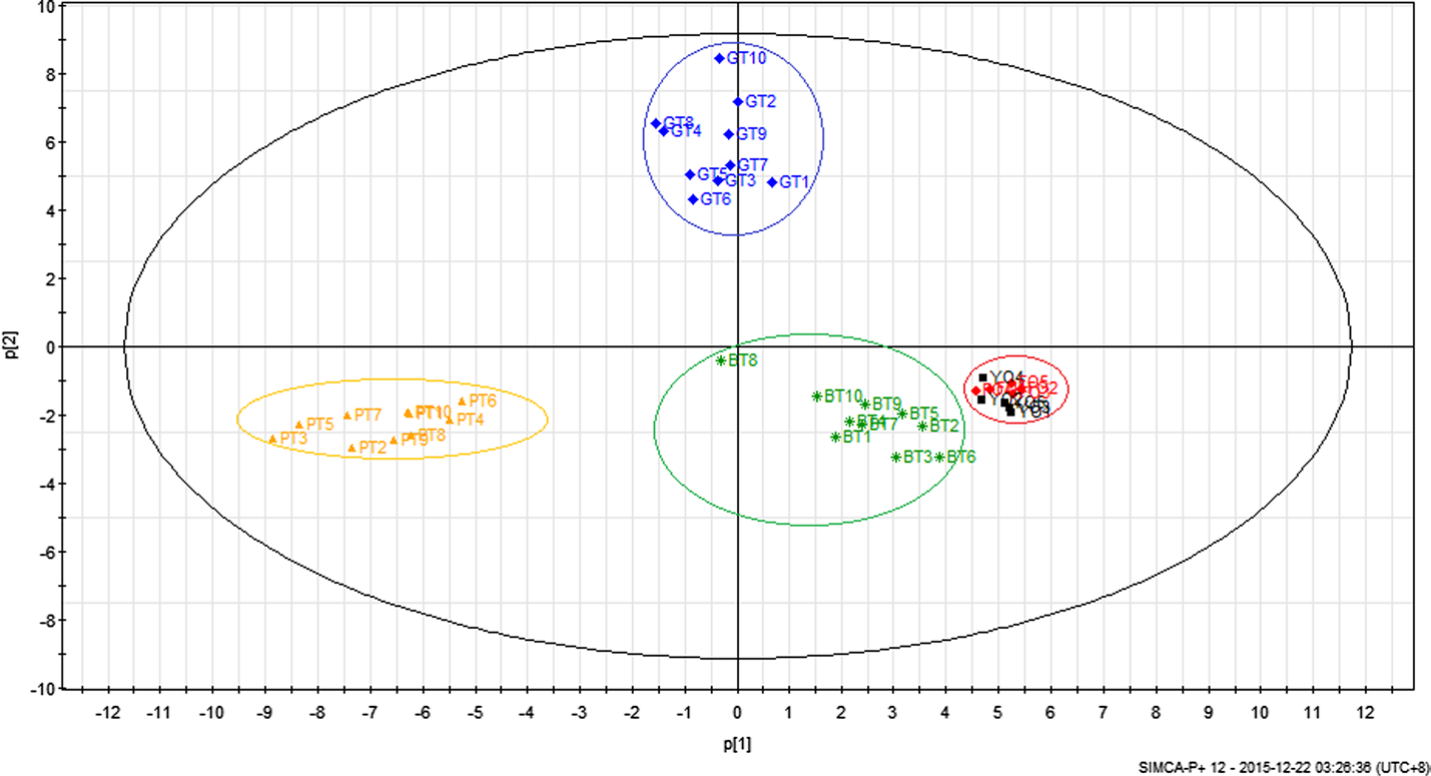
Principal component analysis (PCA) score derived from 92 volatile compounds of 40 tea samples: YO indicated by *black color* represents Yunnan oolong teas, FO indicated by *red color* represents Fujian oolong teas, GT indicated by *blue color* represents green teas, BT indicated by *green color* represents black teas, and PT indicated by *yellow color* represents Pu-erh teas

PCA is an effective way to discriminate between data observed (Ivosev et al. 2008). It also involves a linear transformation of multiple variables into a low-dimensional space that retains the maximum amount of information about the variables (Ma et al. 2013; Wu et al. 2013). Generally, the score plot provides a visual determination of similarity among the samples. PCA (Fig. 4) was conducted with the use of the same data as those used in the CA model. Figure 4 shows that the score plot in the first two principal components (PC1 and PC2) represents 71.43 % of the total variability. The same figure shows that oolong teas (including five Yunnan oolong teas and five Fujian oolong teas) resembled one another closely and were clearly distinguished from the other types of teas in the PCA model; oolong tea was closer to black tea than to the other types of teas. These PCA results were mostly consistent with the results shown in Table 2. The CA and PCA results also suggest that the volatile chemical compounds of the teas analyzed by fully automatic HS-SPME can be used for quality evaluation and control.

**Fig. 5:**
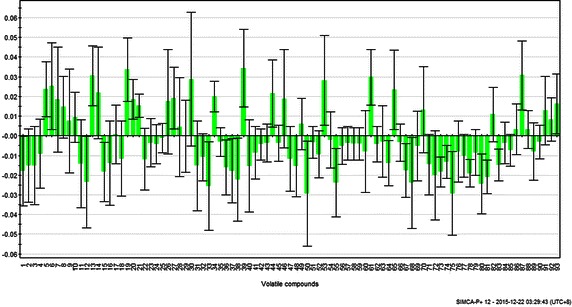
Coefficient plot related to the contribution of 92 volatile compounds to the principal component analysis (PCA) results. The number (No.) of volatile compounds are consistent with that of Table 2

The contributions of all 92 aroma compounds to the PCA results are shown in Fig. 5. The variables that explained maximum variance in the data had high contributions and were considered important in discriminating samples between oolong tea and other types of teas. Benzylalcohol (V13), linalool oxides (V19, V20, V25, and V26), safranal (V30), indole (V39), *β*-ionone (V61), and hexadecanoic acid methyl ester (V87), which contributed to the fruity and flower-like aroma, had high positive values in oolong tea and were thought to enhance their aroma flavor (Kuo et al. 2011). These volatile compounds in oolong tea reached their highest levels during semi-fermentation (Wang et al. 2001). Fermentation was found to lead to the loss of grassy or green flavors and the formation of fruity and other fermented characters (Wang et al. 2008). Some nonalcoholic volatile compounds, such as benzylalcohol, safranal, and hexadecanoic acid methyl ester, were found to be transformed to glycosidically bound forms during fermentation in oolong tea (Guo et al. 1998; Yang et al. 2009). Geranyl pyrophosphate was the precursor for monoterpene alcohols, such as linalool. Some specific terpene synthases are involved in the biosynthesis of volatile monoterpene alcohols, which have been identified and validated in many plants (Creelman and Muleet 1995). Linalool oxide was synthesized from linalool by the possible synthesis pathway of monoterpenoids in tea. And the benzylalcohol in oolong tea was found to be related to the Ehrlich pathway that occurs in fermentation (Bode and Dong 2003). In addition, the tea-derived enzyme in oolong tea plants cleaves the 9,10 (9′10′)-double bonds of arotenoids and long-chained apocarotenoids to yield *β*-ionone (Felfe et al. 2011). This result was also mostly consistent with the typical aroma compounds of oolong tea shown in Table 2. Because the volatile compounds were influenced by biological and chemical transformations during cultivation and processing, we can conclude that these typical aroma compounds, which made oolong tea different from other types of teas, were largely influenced by the semi-fermentation step. Prior to this step, the bruising step breaks the cell membrane and eventually facilitates the mixture of precursors with biological enzymes. Hereafter, the most significant changes are the rapid conversions and transformations of the precursors to benzylalcohol, indole, safranal, linalool oxides, *β*-ionone, and hexadecanoic acid methyl ester, mostly by enzymatic catalysis and chemical processes. Therefore, fermentation intensity influences the quantity of most tea volatiles during the manufacturing process of green, oolong and black tea; and because of the distinctive processes of oolong tea, its aroma characteristics are different from the unfermented green or fully fermented black tea (Baldermann et al. 2014).

In summary, our results suggested that the aroma characteristics of oolong tea, which are either Yunnan oolong tea (*Camellia sinensis* var. *assamica*) or Fujian oolong tea (*Camelliasinensis* var. *sinensis*), were mostly consistent compared with those of the other three types of teas (green, black, and Pu-erh tea). These findings indicated that although the raw materials, cultivation measures used and the environment factors involved in tea production influence water-soluble and aroma components, processing technology plays a crucial role in the formation of tea aroma. Further investigation will focus on the influence of other factors (geographic characteristics, cultivars, etc.), particularly each processing step, on final aroma characteristics in the proposal of guidelines for the quality control of tea products.

## Conclusion

This work reported for the first time that the same types of teas made from different tea tree leaves but the same processing technology showed similar aroma flavor. Our results demonstrated that the sensory evaluation and main water-soluble components, i.e., caffeine, catechins, and total polysaccharides, of Yunnan oolong tea were different from those of Fujian oolong tea, but no significant difference was observed between their aroma characteristics, as shown in the PCA and CA analyses. The PCA results showed that benzylalcohol, indole, safranal, linalool oxides, *β*-ionone, and hexadecanoic acid methyl ester strongly contributed to the aroma flavor of oolong tea compared to the case of the green, black, and Pu-erh teas. Although the raw materials, cultivation measures used and the environment factors involved in tea production influence water-soluble and aroma components among different kinds of teas, processing technique for oolong teas from different tea trees, especially the semi-fermentation process, is the main driver of tea aroma characteristics.
